# Enhancing the Biodiversity of Ditches in Intensively Managed UK Farmland

**DOI:** 10.1371/journal.pone.0138306

**Published:** 2015-10-07

**Authors:** Rosalind F. Shaw, Paul J. Johnson, David W. Macdonald, Ruth E. Feber

**Affiliations:** Wildlife Conservation Research Unit, Department of Zoology, University of Oxford, Recanati-Kaplan Centre, Tubney House, Tubney, Oxon, United Kingdom; University of Hawaii at Manoa, UNITED STATES

## Abstract

Drainage ditches, either seasonally flooded or permanent, are commonly found on intensively managed lowland farmland in the UK. They are potentially important for wetland biodiversity but, despite their ubiquity, information on their biodiversity and management in the wider countryside is scarce. We surveyed 175 ditches for their physical and chemical characteristics, spatial connectivity, plant communities and aquatic invertebrates in an area of intensively managed farmland in Oxfordshire, UK and collected information on ditch management from farmer interviews. Water depth and shade had a small impact on the diversity of plant and invertebrate communities in ditches. Increased shade over the ditch channel resulted in reduced taxonomic richness of both channel vegetation and aquatic invertebrates and channel vegetation cover was lower at shaded sites. Invertebrate taxonomic richness was higher when water was deeper. Spatial connectivity had no detectable impact on the aquatic invertebrate or plant communities found in ditches. The number of families within the orders Ephemeroptera, Plecoptera and Trichoptera (EPT), which contain many pollution-sensitive species, declined with decreasing pH of ditch water. As time since dredging increased, the number of EPT families increased in permanent ditches but decreased in temporary ditches. Whether or not a ditch was in an agri-environment scheme had little impact on the reported management regime or biodiversity value of the ditch. Measures for increasing the amount of water in ditches, by increasing the water depth or promoting retention of water in ditches, could increase the biodiversity value of ditches in agricultural land. Some temporary ditches for specialised species should be retained. Reducing the amount of shade over narrow ditches by managing adjacent hedgerows is also likely to increase the species diversity of plant and invertebrate communities within the ditch. We recommend that to preserve or enhance the biodiversity value of ditches, and improve their ecosystem service delivery, management prescriptions for hedgerows adjacent to ditches should differ from those aimed at hedgerows only.

## Introduction

Where landscapes are dominated by agriculture, semi-natural areas provide critical habitat for biodiversity in an otherwise inhospitable matrix (e.g.[1]). Wetland areas on farmland are increasingly recognised as being important for wildlife, and their loss has been implicated in the decline of some formerly widespread species [2]. Wetland habitats that have been lost in the UK and across Europe include wet rough pasture or marsh, ponds, and small linear features such as ditches [3,4]. Small wetland habitats (both artificial and natural) within agricultural land have been demonstrated to contribute to regional diversity levels [5].

Agri-environment schemes (AES) are currently the most widely applied policy mechanism aimed at halting or reversing the well documented declines of many farmland species [6,7,8]. They provide financial support for farmers and land managers to undertake a range of wildlife-friendly management measures. However, while AES are benefitting some species and habitats [9], others are still faring badly [10]. Given the continuing declines in farmland species, and the cost of AES (for example, approximately £400 million per year is paid to English farmers under AES [11]), improving their effectiveness for biodiversity conservation is a high priority [12]. Recent work has highlighted the need for better assessments of the importance of different habitat features and how to manage them (e.g. [13]).

While a large body of information exists on the biodiversity and management of many semi-natural habitats in farmland, such as field margins and hedgerows, others, particularly small freshwater bodies, remain neglected [14]. Despite having been replaced by sub-surface drainage in some areas [4], ditches are still widespread in agricultural land. As linear wetland areas, they provide very different habitat compared to other farm habitats and are particularly important for aquatic plants [15], aquatic invertebrates [14,16] and amphibians [17]. These groups are also rather poorly studied on farmland, yet contribute to overall species diversity at local and landscape scales [5]. Ditches and their margins may also function as corridors within the landscape for other groups, including pollinators [18] and small mammals [19].

In addition to providing wildlife habitat, ditches supply other ecosystem services such as diffuse pollution mitigation [20]. Both permanent and temporary ditches are often the first concentration point of water draining from agricultural land. The Water Framework Directive of the European Union requires member states to have all surface water bodies in ‘good’ ecological and chemical condition by 2015 [21], and ditches have the potential to reduce the amount of chemicals and sediment entering the wider water catchment [22]. By retaining water across the wider landscape in times of high flow, ditch management may reduce flood risk [22]. Sutherland *et al*. [23] suggest that policy and economic incentives could be used to help maximize achievement of these interlocking objectives: restoring flood plain wetlands, creating riparian corridors, improving in-channel habitat and creating flood retention basins to improve ecological status, reduce flood risk, and deliver biodiversity targets.

The biodiversity of ditches is likely to be affected both by their management and their connectivity to other wetland habitats within the landscape. To maintain their drainage function, ditches require management, particularly removal of vegetation and accumulated sediments to allow water in the ditch to flow. The frequency of ditch management has significant impacts on the vegetation and invertebrate fauna, with intermediate frequency of management commonly having the most positive impacts [24,25,26]. The impacts of management on biodiversity may be mediated by the level of connectivity of the ditch. For example, recolonisation of cleaned ditches may be quicker if they are connected to a source of colonisers (plant diversity of ditch banks has been shown to decrease with distance from a seed source [27]). Environmentally sensitive management of ditches includes reducing the frequency of cleaning and preventing the application of agro-chemicals close to banks [28]. However, some management is usually required, with infrequent management leading to loss of species diversity [25].

Well connected ditches have been shown to have higher species richness (e.g. [27,29]). Temporal as well as spatial connectivity influences aquatic communities. In ditches which dry out regularly, recolonisation can occur from sediments [30], or active or passive dispersal from other areas. Conversely, rapid changes in flow rate in ditches closely connected to main watercourses may have a negative impact on species richness [31]. Connectivity can have implications for the spread of invasive species if, for example, increased connectivity means that water bodies are more likely to be invaded [32].

Much of the information on the biodiversity of ditches has come from permanently wet (e.g. [33,34] ditches or those of high conservation value [25,35,36]. Despite this, small ditches surrounding fields are common in some regions of Europe [4] and North America [37]. In the UK, there are few data on ditches in agricultural land in landscape types other than fenland and river flood plains [38]. Ditches in intensively managed agricultural land are widespread throughout the UK—average UK ditch density is *c*. 2km per km^2^ [38]. The relative lack of information on the biodiversity of smaller, temporary ditches, which are widespread in farmland [14], may lead to lost opportunities for increasing the value of this habitat to farmland biodiversity. This is particularly important as the combined ditch and hedge management option in England’s Entry Level agri-environment scheme (ELS) is one of the most frequently selected. Ditches are eligible for management under ELS only if they contain plants considered typical of wet ditches and farmers receive payments to ensure that these ditches are cleaned less than once per agreement (5 years), and the vegetation cut not more than once every 2 years [39]. In 2009, 43,430km of ditches were managed under ELS [11] and maximising the biodiversity of these is highly desirable.

The aim of this study was to improve knowledge of agricultural ditches using data from a survey of plants and invertebrates in permanent and temporary ditches across multiple farms in intensively managed agricultural land. We investigated the impacts of physical and chemical characteristics, including water quality, the size of the ditch, the bank angle and surrounding land use (which have been demonstrated to be important in other ditch systems e.g. [26,40,41,42]), on biological communities. We also investigated whether management, both vegetation cutting and dredging of the ditch and management of associated features such as hedges, had any effect on ditch communities. Management of ditches can be affected by participation in agri-environment schemes, so we established whether farms or individual ditches were entered into these schemes. We investigated these factors in seasonal and permanent ditches, and in ditches with differing spatial and temporal connectivity across the landscape. We predicted that ditches with a higher proportion of agricultural land surrounding them would have lower biodiversity but that this impact may be mitigated by the presence of associated features such as margins and hedges. We also predicted that ditches with more frequent management would have lower biodiversity but that ditches with greater temporal and spatial connectivity would have greater biodiversity. We also asked whether ditches managed under agri-environment schemes had greater biodiversity than those not in agri-environment-schemes.

## Materials and Methods

### Site Selection

Ditches were defined as man-made channels, primarily for agricultural purposes which usually have a linear planform, follow linear field boundaries, and show little relationship to natural contours (following [16]). The study area was the Upper Thames region (northern extent 51.980024°, -1.2789423°, southern extent 51.6642°, -1.495128°, eastern 51.690568°, -1.6601901°, western 51.716468°, -0.96372977° DD) which represents pre-quaternary clay landscapes with some river floodplain areas [38]. Farms were randomly selected using postcodes (http://www.yellowpages.com/) to obtain farmer contact details. Sixty-five farmers were contacted. Of these, 18% did not have ditches and 11% did not want to participate, resulting in 175 ditches from 30 farms being included in the survey. Ditches included in the study ranged from narrow temporary ditches, to large ditches which contained water all year (see Table 1 and Table 2). All ditches had been dug as drainage ditches and most were more than 45 years old.

**Table 1:** Physical and chemical characteristics of surveyed ditches.

Characteristic	Mean (± SD)	Range (n)
Ditch length (m)	288.4 ± 166.23	28–1292 (175)
Bottom width (m)	0.98 ± 0.564	0.25–4.5 (175)
Bank top width (m)	3.49 ± 1.55	0.9–11 (175)
Bank angle (degrees)	41.1 ± 9.47	9–80 (349)
Water depth (m)	0.07 ± 9.77	0–1 (175)
Freeboard (m)	0.84 ± 0.34	0.03–1.65 (175)
pH	7.9 ± 0.43	6.04–9.05 (270)
Conductivity (μS)	858.00 ± 410.30	210–3924 (294)
Nitrate (mg/L nitrate-nitrogen)	3.02 ± 3.47	0–17.5 (243)
Phosphate (mg/L)	2.01 ± 3.46	0.01–29.15 (245)
Number of spatial connections	3.64 ± 1.82	0–10 (173)
Amount of shade (%): Ditch bank with hedge	61.1 ± 29.5	0–100 (129)
Amount of shade (%): Ditch bank without hedge	15.8 ± 24.7	0–90 (192)
Amount of shade (%): Ditch channel with hedge	38.1 ± 32.2	0–95 (141)
Amount of shade (%): Ditch channel without hedge	21.1 ± 31.5	0–90 (31)

**Table 2:** Connectivity, land use and management of ditches. Participation in agri-environment schemes (AES) includes Entry Level Stewardship Schemes (ELS), Higher Level Stewardship Schemes (HLS) and Environmentally Sensitive Areas (ESA) (http://www.naturalengland.org.uk/ourwork/farming/funding/default.aspx)

Characteristic	Category	Number of ditches
Temporal connectivity (from farmer interviews)	Wet after heavy rain	17
	Winter wet and wet after heavy rain	59
	Winter wet	37
	Wet all year	61
Land use surrounding ditch	Predominantly arable	71
	Predominantly pasture	63
	Mixed (arable, pasture)	33
	Woodland	5
Number of ditches with associated features	Hedgerow	154
	Field margin (≥3m) on one side	63
	Field margin (≥3 m) on both sides	41
Participation in AES (numbers of farms with ditch option selected in parentheses)	ELS	19 (6)
	HLS	3 (1)
	ESA	1 (0)
	NO AES	7

### Ditch selection and survey

For recording local factors such as aspect and immediate land use, ditches were defined as ending at a junction with another ditch, other linear feature (e.g. hedgerows), or drainage pipe. The number of ditches surveyed per farm ranged from 1 to 14, (mean 5.8 ± 2.77 SD). If farms contained fewer than six ditches, all were surveyed. If the farm had more than six ditches, ditches were selected at random (if they appeared homogenous) or selected to cover the range of ditch types on that farm. All ditches were surveyed for physical and chemical characteristics, and bank and channel plant communities (see below). A subset of ditches that were at least partially wet at the time of survey was sampled for aquatic invertebrates.

### Physical and chemical characteristics of ditches

Ditches in our study differed from each other in terms of length and width, which could affect their biodiversity. We therefore measured ditch length, bottom width and bank top width and used the formula for a trapezoid area multiplied by the length to estimate ditch volume (ditches tend to be more consistent than natural watercourses in channel shape).

Bank angle was estimated using a clinometer. A spatial connectivity index was calculated (number of spatial connections at the end of each ditch multiplied by a qualitative score for each connection type: 0 = earth bank, 1 = connected by a culvert or pipe to another ditch, 2 = connected directly to another ditch, stream or river). Although coarse, this method was used because mapping the number of ditches within a radius of the study ditch might not represent whether the study ditch was actually connected to any of the other ditches in the network and, furthermore, minor ditches are not always mapped. Each ditch was assigned to a temporal connectivity category based on the reported perception of the land manager. The categories recognised were wet all year (permanent), wet during winter and after heavy rain, wet during winter, or wet after heavy rain. Presence and width of uncropped or fenced field margins (alongside the ditch and abutting the ditch bank) were recorded, together with the percentage of each bank length with a hedge. The percent shade from woody vegetation was estimated as the percentage of bank or channel that would be shaded if the sun was directly overhead, during the summer surveys (i.e. with full foliage). Water depth, pH and conductivity were recorded on each of three visits (summer 2010, winter-spring 2011, summer 2011) and averaged before inclusion in analysis. The pH and conductivity were measured in the field using a multiparameter tester (Hanna Instruments, UK), and water samples were taken, frozen within 8 hours and analysed for nitrate (cadmium reduction method) and phosphate (ascorbic acid method), on an environmental bench photometer (Hanna Instruments, UK). Samples were tested in duplicate. If two samples gave readings that differed by >10% it was considered that there was a replication error and the analysis was repeated. The mean result from the three sampling periods was used in the analyses.

### Local land use and ditch management

Farmers participating in the ditch survey were interviewed in person between February and April 2011. Farmers were asked if they had an AES agreement, and whether it included options related to ditch management. For each ditch, farmers were asked: its age and purpose; if the ditch was managed under an AES; when the ditch vegetation was last cut; when the ditch was last dredged; and if any other management had been applied. For adjacent hedges, the time since cut was recorded. S1 Table gives full details of the questions asked. Land use (arable, here defined as annual crops; pasture, both temporary leys and permanent grassland; woodland or other) on either side of the ditch was recorded in the field, following Palmer *et al*. [43].

### Vegetation surveys

Vegetation surveys were conducted in the summers of 2010 (August-Oct, 86 ditches) and 2011 (late July-Sept, 87 ditches). Percentage cover of higher plant species (following [44]), mosses, liverworts, bare ground and plant litter was estimated by eye along the middle 10 m length of each ditch bank (sloping sides of ditch, above water line if wet) and channel (aquatic or dry base of ditch) using a 10 m measuring tape as a guide.

### Invertebrate surveys

Invertebrate surveys were carried out between May and early July 2011. Sweep netting and timed bank sorting were carried out following the methods of Palmer *et al*. [43]. Sweep netting was also timed (for three minutes) for consistency with Environment Agency methods [45]. Invertebrates were stored in 90% ethanol and identified by APEM Ltd (Stockport, UK), according to Palmer *et al*. [43]) This method involves identifying key groups to species (all aquatic groups excepting Diptera larvae, Oligochaeta and Bivalvess). The number of Ephemeroptera, Trichoptera and Plecoptera (EPT) families per ditch were recorded. Many EPT families are pollution sensitive and used for quality assessment of still waters such as ponds and canals [46]. Invertebrate abundance was recorded on a logarithmic scale (1 = 1–9, 2 = 10–99, 3 = 100–999, 4 = 1000+ [43]).

### Statistical analysis

Several measures were used to assess the biodiversity value and habitat quality of ditches: i) taxonomic/ species richness for invertebrate and plant channel communities (for bank vegetation data, the Berger-Parker Index [47] was used as a proxy for taxonomic diversity due to differences in bank width potentially biasing other metrics); ii) habitat quality score of Palmer *et al*.[43] for aquatic plant communities (based on Ellenberg nitrogen indicator values, and indicating the water quality of ditches); iii) number of Ephemeroptera, Trichoptera and Plecoptera (EPT) families per ditch; iv) community assemblages.

The effects of physical and chemical characteristics, spatial connectivity and land use on ditch biodiversity were tested using a set of models based on plausible hypotheses developed from the literature (see S2 Table). These hypotheses led to the framing of both main effects models to represent factors such as ditch size or the presence of hedge and also models including plausible interaction effects such as those including surrounding land use and the presence of a vegetative field margin. Biodiversity indices were analysed using mixed effects models with maximum likelihood [48] using R software [49]. Models were constructed using Site ID as a random effect to account for similarity among ditches at the same farm. For invertebrate taxonomic richness an offset of the length of ditch sampled was used to account for the survey length being less than 50m in some ditches. Before model fitting, samples with missing values for any predictors were removed. The number of EPT families per ditch was analysed using a generalised linear mixed model with a Poisson distribution (package lme4 [50]). Residuals were checked for normality and the habitat quality score log transformed to an approximately normal distribution.

Model selection procedures [51] were carried out using Akaike’s Information Criterion (AIC) adjusted for small samples (AICc) using R package MuMIn [52]. Models with AICc values that were less than 7.0 greater than that of the best model were considered plausible, provided they were a better fit than the null model [51]. Model averaging was not used due to correlated explanatory variables leading to inflated estimates of parameter standard error. The amount of variation explained by each model was assessed using a likelihood-ratio based pseudo R sq [52] and Rsq adj following Nagelkerke [53]. Community analysis was carried out using the R Vegan package [54]. Continuous environmental variables (same as those used in taxonomic richness analysis, see S2 Table) were standardized to zero mean and unit variance and response data were Hellinger transformed [55]. Partial redundancy analysis (pRDA) was used to partition variation explained due to explanatory variables, and variation due to the covariables site (farm) and, for vegetation data, quadrat size [56]. These covariables were treated by the pRDA as ‘conditioned’ variables, with variance explained by these removed, in the multivariate equivalent of a partial regression analysis [55]. For banks, one bank from each ditch was randomly selected for analysis. The pRDA was carried out using the factors included in the taxonomic richness models but without interaction terms. The global model was tested for significance using 999 permutation tests and, if significant, forward stepwise selection using double-stopping criteria was used to select the final model [57]. The Rsq adj were calculated using variance partitioning [58], which aims to separate the variation explained into that explained by the different sets of variables (environmental and conditioned). As the two explanatory datasets are not orthogonal to one another (the variables are inter-correlated), some variation is explained jointly by the explanatory and conditioned variables [55].

The models above suggested both shade and water depth were important environmental predictors. Reasoning that these were plausibly affected by hedgerows, we used them as responses in models with average interval of hedge cutting by the manager and the amount of hedge next to the ditch as predictors using linear mixed effects models (LMMs, R package nlme [48]). Site was entered as a random effect and the average water depth was square root transformed to meet the model assumption that data are normally distributed. The effect of AES status on ditch management (time since dredged and time since vegetation cut) was tested using general linear models with a Poisson distribution. As the majority of sites had only ditches that were managed under AES or ditches that were not managed under AES, site was entered first in the model to account for this variation.

### Ethics Statement

All fieldwork was carried out on private land with permission of the land owner. No protected species or vertebrates were sampled as part of this project. Data collected from farmer questionnaires were anonymised prior to analysis, and informed written consent was received from all questionnaire participants. This study was approved by Oxford University Central University Research Ethics Committee.

## Results

### Factors affecting ditch vegetation

#### Bank vegetation

**Fig 1:**
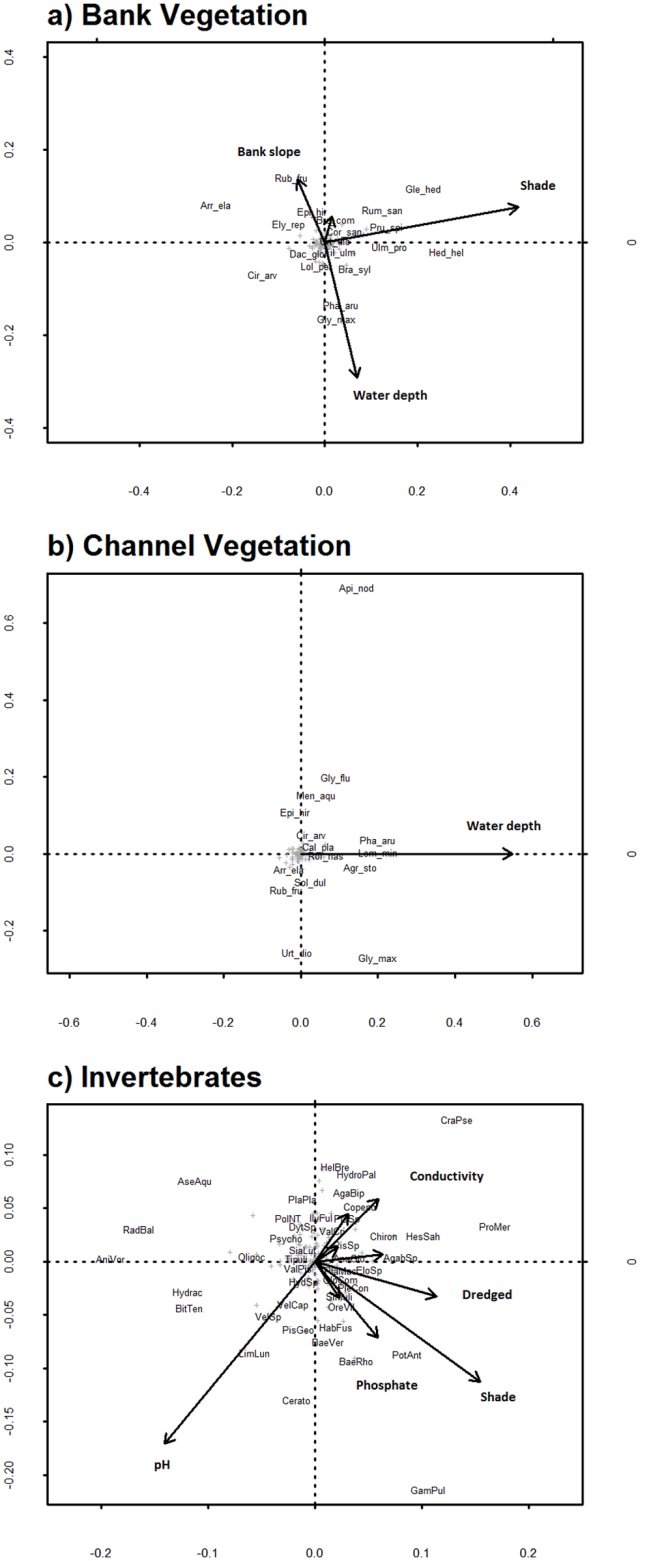
Correlation biplots from the final partial RDA models showing species and explanatory variables for a) bank vegetation b) channel vegetation and c) aquatic invertebrates. In a) shade was entered as a quadratic term. Species codes consist of [first 3 letters of genus name]_ [first 3 letters of species name], see S1 Dataset for full taxonomic names.

One hundred and fifty-four ditches were included in the ditch vegetation analyses. Between 2 and 39 plant species were recorded on ditch banks (mean 12.5 ± 5.94 SD). None of the predictor variables explained any of the variation in the Berger Parker index. The vegetation community composition was most strongly affected by shade and the water depth (Table 3), however the amount of variation explained by the environmental variables was low (Table 4). The multivariate analysis showed that shaded ditches had a higher abundance of woodland and hedge ground cover species, such as *Hedera helix* and *Glechoma hederacea* (Fig 1a). Increased water depth was associated with the presence of species such as *Glyceria maxima* and *Phalaris arundinacea*.

**Table 3:** Results from final model in partial redundancy analyses of community data using the final model selected, using forward stepwise selection with double stopping criteria (Blanchet *et al* 2008). Significance of each term in the final model was tested using 999 permutation tests: a type I test (in sequential order) and type III test (test of marginal significance).

Community			Type 1 test		Type III test	
	Final pRDA model	Df	Variance	P	Variance	P
Bank vegetation	% shade	1	0.024	<0.001	0.023	<0.001
	Water depth	1	0.011	0.012	0.011	0.01
	Ditch in AES	1	0.006	0.420	0.006	0.448
	Bank slope	1	0.008	0.113	0.008	0.087
Channel vegetation	Water depth	1	0.008	<0.001	0.008	0.005
Aquatic invertebrate	pH	1	0.027	<0.001	0.021	0.015
	Water depth	1	0.015	0.091	0.012	0.390
	Dredged	1	0.015	0.091	0.018	0.020
	Conductivity	1	0.018	0.028	0.0126	0.054
	Phosphate	1	0.016	0.054	0.012	0.240
	% shade + % shade sq	1	0.020	0.006	0.022	0.010
	% arable	1	0.013	0.172	0.013	0.200
	Ditch in AES	1	0.013	0.294	0.012	0.310
	Nitrate	1	0.013	0.166	0.013	0.320

**Table 4:** Amount of variation explained by environmental (Env) and conditioned (Site and Quadrat Size) variables in the partial redundancy analyses of the bank vegetation community, channel vegetation community and aquatic invertebrate community (see Table 3 for environmental factors included in final models, conditioned variables were always site and quadrat). Conditioned variables are treated as covariates and held constant whilst investigating the amount of variation accounted for by explanatory variables. Some variation is explained jointly by environmental and conditioned data sets (cannot be attributed to either data set) as they are not orthogonal [55].

	Bank vegetation community	Channel vegetation community	Aquatic invertebrate community
Env adj	0.07	0.04	0.11
Site adj	0.07	0.10	0.08
Env + Site joint variation	0.02	0.01	0.04
Quadrat Size	0.004	0.01	NA
Unexplained variation	0.86	0.88	0.77

#### Channel vegetation

**Fig 2:**
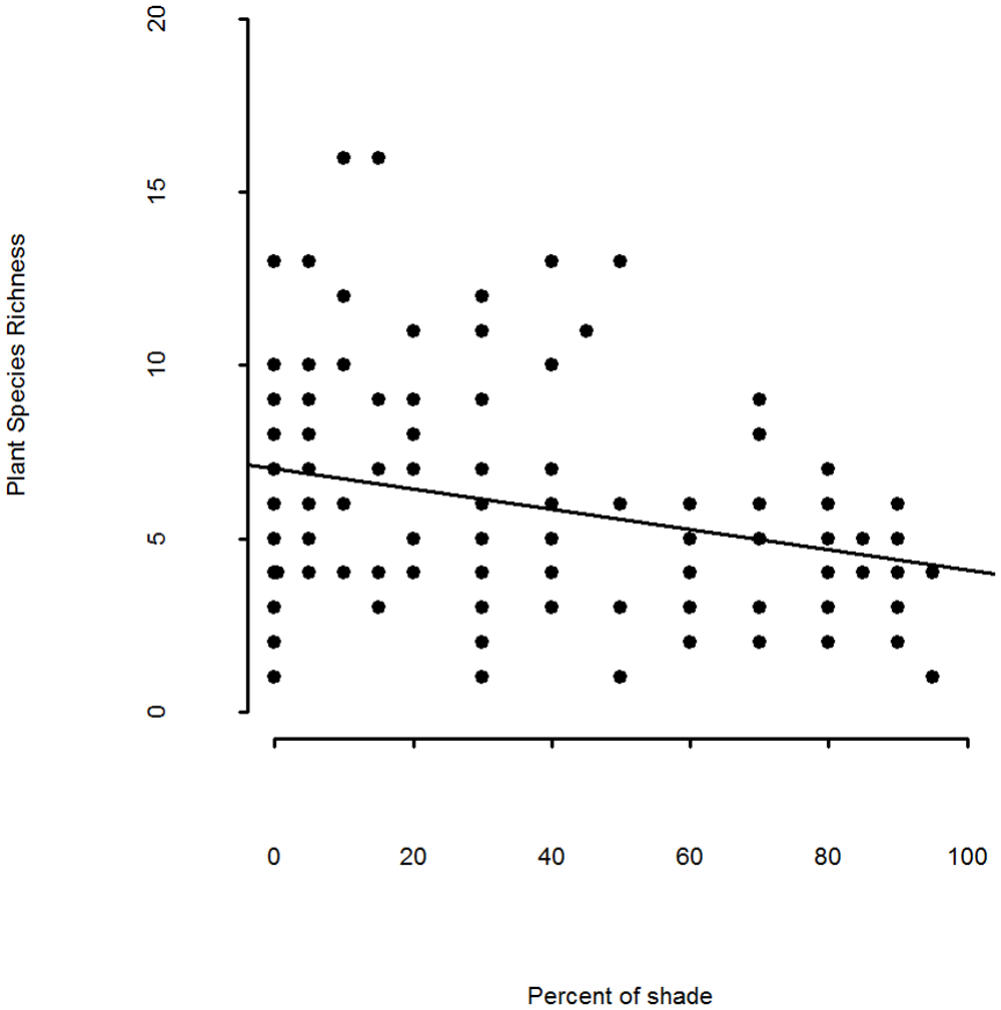
Species richness of aquatic plants per 10 m length of ditch bank plotted against percentage shade over ditch channel. Regression line fitted from a simple linear model of predictor plotted against response without random effects.

The number of plant species found in the ditch channel varied between 1 and 15 (mean 5.4 ± 3.10 SD). The best fitting model demonstrated that greater shade was associated with reduced plant species richness (parameter estimate -0.026 ± 0.0072 SE, Fig 2, Table 5). The second best fitting model included the amount of shade as a quadratic term. The amount of variation explained by all variables was low (global model Rsq adj 0.19, see S2 Table for variables included), as was the amount of variation explained by the highest ranked model (Rsq adj 0.08, Table 5). The mean Habitat Quality Score of wet ditches was 1.3 ± 0.33 SD. There was no evidence for any impact of the predictor variables tested on the habitat quality score.

**Table 5:** Model selection table from analyses with response variables i) Channel vegetation species richness; ii) Invertebrate taxonomic richness and iii) Number of Ephemeroptera, Plecoptera and Trichoptera (EPT) families per ditch. Only models which were a better fit than the null model are listed.

Response variate	Model	(Intercept)	df	logLik	AICc	delta	weight	Rsq	RsqAdj
i) Channel plant species richness	Percent shade over channel	7.28	5	-376.31	763.02	0	0.53	0.08	0.08
	Percent shade over channel + Percent shade over channel squared	7.10	6	-375.40	763.38	0.36	0.44	0.09	0.09
	Percent arable _*_Percent hedge_*_total margin width	8.47	11	-373.91	771.68	8.66	0.007	0.11	0.11
ii) Invertebrate taxonomic richness	Average water depth	14.416	5	-124.795	261.257	0.000	0.422	0.178	0.179
	Percent shade over channel	16.802	5	-126.119	263.904	2.646	0.112	0.125	0.125
	Percent arable	14.188	5	-126.533	264.733	3.475	0.074	0.107	0.108
iii) Number of EPT families per ditch	Average pH	-16.67	4	-30.45	69.88	0.00	0.52	0.26	0.33
	Dredged_*_Temporal connectivity	-0.75	6	-28.12	70.39	0.51	0.40	0.33	0.41
	Time since dredged	-1.72	4	-34.23	77.44	7.56	0.01	0.13	0.16
	Post hoc model −pH + Dredged_*_Temporal connectivity	-10.35	7	-25.59	68.12	0	0.56	0.40	0.50

The channel vegetation community composition was affected by water depth (Fig 1b). The amount of variation explained by the final model was very low (Rsq adj 0.04), as was the amount explained by site (Table 4). Ditches with greater water depths were associated with species more characteristic of open water such as *Lemna minor*.

### Aquatic invertebrates

**Fig 3:**
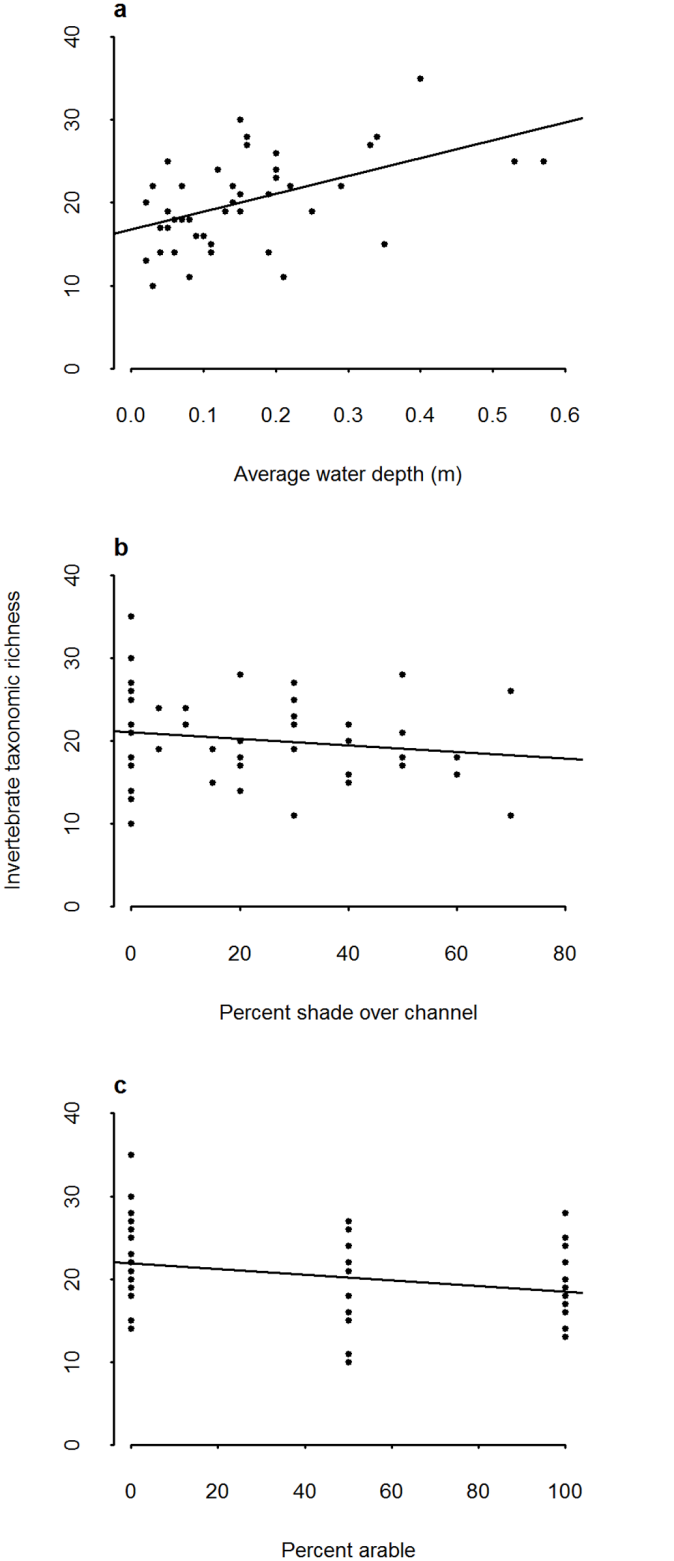
Invertebrate sample taxonomic richness in relation to a) average water depth b) percent shade over the ditch channel and c) mean taxonomic richness in relation to percent arable land in the surrounding fields. Regression lines are fitted from a linear model of predictor plotted against response without random effects.

**Fig 4:**
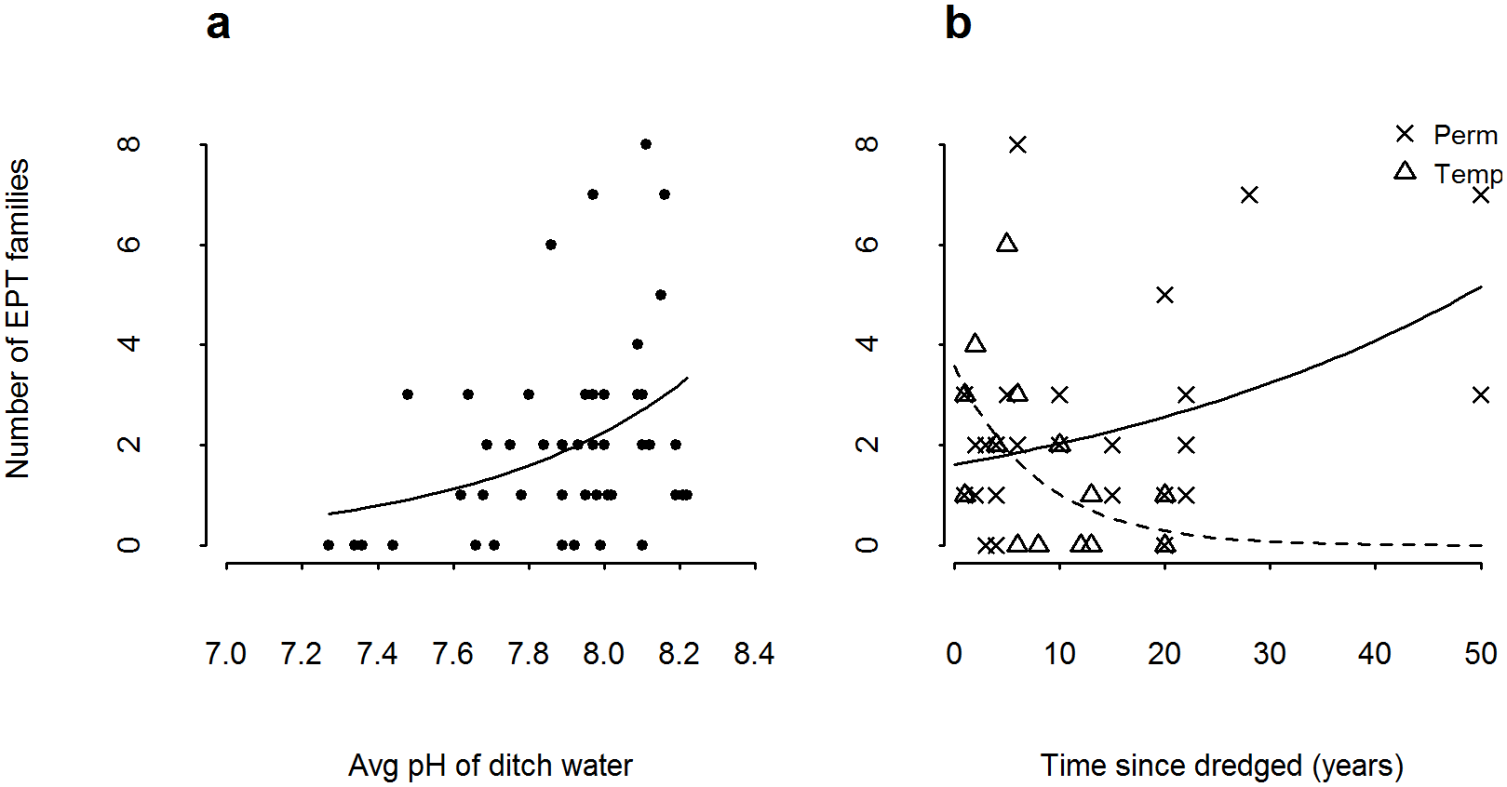
Number of Ephemeroptera, Plecoptera and Trichoptera (EPT) families per ditch in relation to a) pH and b) temporal connectivity of ditch and time since dredged (black circles and line, permanent ditches, open triangles and dashed line temporary ditches). Error bars (c) are 95% confidence intervals. Regression lines are fitted from a generalised linear model with poisson distribution of predictor plotted against response without random effects.

Of the surveyed ditches, 49 were wet at the time of the aquatic invertebrate survey. From these ditches, 190 invertebrate taxonomic groups were identified, and taxonomic richness varied between 9 and 35 per ditch (mean 19.6 ± 5.76 SD). The ditches surveyed did not cover the full range of temporal connectivity (there was only one ditch which the farmer considered to be winter wet only, and no ditches categorised as wet after heavy rain were wet at the time of survey). Ditches were therefore grouped into permanent ditches (wet all year) and temporary ditches (all other categories). The model with the best fit to the data contained average water depth (Table 5) indicating that taxonomic richness increased with water depth (parameter estimate 19.9 ± 6.14 SE, R sq adj 0.179, Fig 3a). The second best fitting model had considerably lower weight than the first and included percent of the channel shaded (parameter estimate -0.09 ± 0.037 SE, R sq adj 0.125, Table 5, Fig 3b). The extent of arable land surrounding the ditch had a small negative impact on taxonomic richness (parameter estimate -0.04 ± 0.019 SE, Rsq adj 0.11, Fig 3c). The number of Ephemeroptera, Plecoptera and Trichoptera (EPT) families per ditch was positively associated with increasing pH of the ditch water (parameter estimate 1.9 ± 0.58 SE, Table 5, Fig 4a). A very similar model in terms of fit and explanatory power included temporal connectivity and time since dredged. Temporary ditches had lower numbers of EPT families as time since dredging increased, whereas permanent ditches had higher numbers of EPT families as time since dredging increased (parameter estimate interaction term 0.13 ± 0.04 SE, Fig 4b). A post-hoc model including terms from the best two models had an Rsq adjusted of 0.50 (Table 5).

The global model for the invertebrate community analysis had an Rsq adj of 0.15. Variance partitioning indicated that the variation explained solely by the environmental variables selected using forward selection was 0.11 (Rsq adj), while the additional variation explained by these variables but which could not be attributed to either owing to their inter-correlation was 0.04 Rsq-adj. Three factors were statistically significant predictors of the invertebrate community (Table 3, Fig 1c): pH, conductivity and the amount of shade entered as a quadratic term. Species associated with higher pH included *Limnephilus lunatus* (caddisfly larvae), *Velia* spp (riffle bugs) and Succineidae (amber snail slugs). Those associated with higher shade included *Potamopyrgus antipodarum* (Jenkins spire snail shell), an invasive species in the UK, native to New Zealand (Fig 1a).

### Effects of management on influential environmental predictors

Eighty-eight percent of the ditches in this study had a hedge on one or both banks (Table 1). Ninety-four percent of hedges on ditch banks were sited directly at the top of the ditch bank. On the remaining 6% the hedge was, on average, 0.68m (± 0.181 SE) away from the top of the ditch bank. Ditches with a lower proportion of hedge along the bank had lower levels of shade over both the channels and banks (parameter estimate 0.27 ± 0.057 SE, F_1,175_ = 22.9, *P*<0.001 for channel data, 0.51 ± 0.030 SE, F_1,294_ = 285.6, *P*<0.001 for bank data). There was no relationship between the frequency of hedge trimming and the amount of shade either for individual banks (F_1,79_ = 0.22, *P* = 0.64) or when assessed across the whole channel (F_1,74_ = 0.27, *P* = 0.60). There was a negative relationship between the percent of banks with hedge present and the average water depth (parameter estimate -0.001 ± 0.0002 SE, F_1,125_ = 16.2, *P*<0.001). Increased shade over the channel resulted in a reduction in the percent cover of vascular plant communities (parameter estimate -0.57 ± 0.069 SE, F_1,139_ = 67.4, *P*<0.001).

### Management of ditches under AES

At three sites, all ditches surveyed were included in an AES, 21 sites had no ditches included in an AES and 5 sites had some ditches managed under an AES and some not. One land manager could not tell us which ditches were managed under an AES agreement. Site (i.e. individual farms and therefore farmers as a fixed effect) had a strong effect on the time since last dredged and since the last vegetation cut. On sites with both AES and non-AES ditches, ditches in AES had been dredged less recently than those not in AES (Table 6, parameter estimate -0.68 ± 0.164, z = -4.161, p <0.001). There was no significant difference in the time since the vegetation was last cut between AES and non-AES ditches, once variation for site had been accounted for (parameter estimate -0.03 ± 0.180, z = 0.161, p = 0.87).

**Table 6:** Management of ditches in agri-environment schemes (AES) in England (Entry Level Stewardship, Higher Level Stewardship, Environmentally Sensitive Areas, Countryside Stewardship Scheme [38]) and those not in AES. Data in square brackets beneath are ditches from sites where land managers had both ditches that were managed under AES and those managed outside of AES.

Management	Ditch in AES		Ditch not in AES	
	Mean (years) ± SD	Range (n)	Mean (years) ± SD	Range (n)
Time since last dredged	14.7± 14.55	1–37 (32)	16.0 ± 14.74	1–55 (122)
	[10.9 ± 8.00]	[1–28 (25)]	[6.2 ± 8.12]	[1–28 (12)]
Time since vegetation last cut	3.7 ± 1.82	1–10 (32)	9.4 ± 13.59	0–50 (122)
	[5.3 ± 5.15]	[1–20 (25)]	[6.2 ± 8.12]	[1–28 (12)]
Interval of hedge cutting	2.9 ± 2.35	1–10 (23)	3.75 ± 5.33	1–22 (84)
	[3.7 ± 3.09]	[1–10 (13)]	[2.2 ± 1.79]	[1–5 (5)]

## Discussion

Numbers of plant species per ditch were lower in this study than those reported from coastal grazing marshes in England and Wales [26], but similar to those reported from a study of ditches across Europe [59]. The Habitat Quality Score of aquatic plant communities in our study was similar to that found in a study of coastal grazing marsh ditches (1.3 in this study, compared to 1.7 in Drake *et al*. [26]). The biodiversity of the invertebrate community was similar to that found in temporary agricultural ditches in Maryland [60] and permanent ditches across Europe [59].

The amount of variation explained by the environmental factors tested was low for most groups. However the amount of shade over the ditch and the water depth had a small but significant effect in several analyses. Both can be influenced by management. Bank vegetation, when shaded, was dominated by plants typical of woodland communities. The species richness of channel vegetation decreased as the amount of shade over the channel increased. Contrary to expectation, the reported interval between hedge cutting was not related to the amount of shade over the ditch. Two possible reasons for this are, first, that hedges with trees may be less frequently managed but result in patchier (overall lower) levels of shade than frequently trimmed dense shrubby hedges and, second, only the hedge side furthest from the ditch may have been cut (due to restricted access, and to prevent trimmings falling into the ditch). While large amounts of hedge trimmings in ditches are considered undesirable, as they may lead to blockage of ditches during periods of high flow [61], a small amount of woody vegetation in ditches may benefit biodiversity by increasing habitat heterogeneity [62].

Increased water depth had a positive impact on both plants and invertebrates, with more plants considered characteristic of ditches on the banks of wetter ditches (making the ditch eligible for AES funding [39]). Taxonomic richness of both channel vegetation and invertebrates was greater in ditches with greater water depth. The positive impact of water depth on taxonomic richness may have been influenced by the exceptional dryness of the previous year (2009–2010). However, other studies have found that increasing water depth increases ditch vegetation diversity [63] and the abundance of emergent insects [64]. Twisk *et al*. [63] found increasing the water depth was the most cost effective way of improving the diversity of ditch vegetation, although, in water depths above 50–70cm, plant species richness declined due to light limiting conditions. This agrees with our results as, although we found no evidence of a decline, the depth of our deepest surveyed ditch was only 57cm. Deeper ditches are potentially less vulnerable to temperature fluctuations [65], or may have lower nutrient loads [66].

Water depth in ditches can be increased by installing dams or barriers, which can be relatively low cost [22,67]. Recent increased interest in rural sustainable drainage systems indicates that increasing water retention in ditches may have multiple benefits, including mitigating water pollution and retaining water in times of high flow to help prevent flooding in the wider landscape [22]. Previous studies have found no link between species richness and flow control structures, although they may alter invertebrate communities that are highly influenced by flow rates [60]. The implications of alterations of water flow for flooding potential in the fields directly around such features need to be considered: a short study of bunded ditches fitted with overflow pipes found no reduction in yield in the surrounding fields, however it was only assessed in one year [67]. Our research suggests that such schemes may have the potential to increase the biodiversity value of ditches. However, we note that some seasonal ditches may sustain uncommon temporary water invertebrate species that may not be present in any other (permanent) water body type [16]. A further consideration is that increases in water depth, and also reduction in shade, may lead to increases in dominant plant species and eventually result in a decline in biodiversity levels if they are not removed by management [26,63]. Allowing ditches to become shaded may reduce the required frequency of management, but lead to relatively low biodiversity levels [61]. Increasing the water depth and reducing shade may lead to more rapid succession, thus requiring more frequent disturbance through management. This may result in higher biodiversity levels ([68,69] but see [70]). The current recommendations for maintaining ditch biodiversity by using a variety of disturbance levels derive mainly from wet ditches (e.g. [26]) but would apply equally to more temporary ditches if their water depth was increased.

Spatial connectivity of ditches appeared to have little impact on the invertebrate or plant communities found in them; the index of spatial connectivity may have been too coarse to detect any effects. Temporary ditches were found to have lower numbers of EPT families with time since dredging, whereas the opposite was true for permanent ditches. The number of EPT families is used as indication of the pollution level of water bodies, with more EPT families indicating lower pollution levels. In permanent ditches, dredging is likely to lead to increased sediments in the water and reduced uptake of any pollutants entering the ditch [20,71], which may result in lower numbers of EPT families in ditches as they recover from disturbance. Temporary ditches appeared to have an opposite relationship. The build-up of sediment in temporary ditches due to potentially lower flow rates, and less ‘flushing’ of the ditch, may mean that ditches which have not been dredged for a long time contain more agrochemical residues. The presence of EPT families is unlikely to be limited by dispersal, due to the mobility of the adult stages.

Land use surrounding the ditch affected the species richness of the invertebrate community, with lower invertebrate taxonomic richness in ditches entirely surrounded by arable land. Ditches closer to nature reserves (the closest bordering nature reserves) in the Netherlands had greater species richness [40], but relatively few studies compare ditches in pasture and cropped arable land [72]. Ditches in arable land are likely to receive higher pesticide load than pastures [38], as well as different nutrient inputs and sediment run off [73]. Agri-environment schemes have, in part, been developed to mitigate for the effects of intensive agriculture. However, there was little evidence from this study that the biodiversity of a ditch was affected by management under an AES agreement, as has also been demonstrated for other AES schemes in Europe [74,75]. There was no difference between vegetation cutting regimes in AES and non-AES ditches in this study (Table 6). Boatman *et al*. [76] found 30% of ELS farmers in England had to reduce the frequency of bank vegetation cutting to meet AES requirements, but only 8% needed to reduce the frequency of dredging. While we found that ditches in AES had been dredged less recently than non-AES ditches on the same site, the mean time since non-AES ditches had been dredged was approximately 6 years. An AES agreement in England currently runs for 5 years, which suggests that a number of these farmers would not have to have reduced the dredging frequency (similar to [76]). Overall, management in this study appeared to be less frequent compared to that recorded for high conservation value ditches in fenland (dredging cycles of 1–4 years and 4–10 years for the Somerset Levels and Broads ESA respectively [77]). Currently, to be eligible for inclusion in AES, ditches are required to contain typical ditch plant communities [39]. In contrast to other linear habitats, such as field margins and hedgerows, which can be created or restored with AES support, there is no provision within the lower tier AES in England for improvement or restoration of ditches [39]. We suggest that, given their importance as habitat for different communities within the landscape, a higher priority should be given to creation and management of small wetland features, such as ditches, within AES.

## Conclusions

Our data suggest that many farm ditches are underperforming in terms of delivering biodiversity benefit, but this could be improved with relatively simple changes in management. Provision of AES options that would allow currently ineligible ditches to be improved would fill one gap. For example ditches without characteristic hydrophilic plants are not eligible for support under AES, but might benefit from directed management to increase the amount of water or reduction of heavy shade from associated hedges. Simple measures such as barriers to increase the amount of water in the ditch may also be beneficial, if appropriate to the ditch and surrounding area [22] and fitted with overflow pipes in case of heavy rain (see [67]). Currently, ecosystem service benefits from a ditch which has a hedge next to it are assumed to be the combined impacts of hedges and ditches [78]. The current English AES framework has existing separate options for ditch management alone, or ditch and hedge combined management [39]. The combined management option for ditches and hedges is similar for these features when they occur on their own. The combined option recommends that hedge trimmings should not be allowed to fall in the ditch [39]. However, these management options currently do not include information about reducing over-shading from the hedge next to the ditch. Hedges which create a high level of shade over the associated ditch could be cut on the side next to the ditch. The increase in light available to plant communities may result in increased plant biomass and this may lead to increased sedimentation as demonstrated in saltmarsh communities [79]: this may lead to a need to increase the frequency of ditch management to maintain their drainage function. However, this cost to the land manger may be offset by the wider benefit of reduced sediment and potentially agricultural pollutants in water leaving farmland, leading to an improvement in both the biodiversity value of ditches and improvement in the quality of surface waters.

## Supporting Information

S1 Dataset Excel file of 6 datasets used in the analyses. (XLSX)

S1 Table Farmer interview questions. (DOCX)

S2 Table Full list of models used in the model selection procedure. (DOCX)
