# Supplementary material for: Enhancing the Biodiversity of Ditches in Intensively Managed UK Farmland
Source: PLoS One. 2015 Oct 7;10(10):e0138306. doi: 10.1371/journal.pone.0138306 (PMC4596843; doi:10.1371/journal.pone.0138306)
Supplement: S1 Table — (DOCX) [file pone.0138306.s002.docx]

Table S1. Questions asked of land managers as part of in person interview about ditch management. The interviewer had a map of ditches and, when questions about specific ditches were asked, they were identified on the map to the participant.

| Question | Notes |
| --- | --- |
| 1. How long have you managed/owned this farm? |  |
| 1. What is your main farming enterprise? | Farmers were given the options “Arable”, “Livestock”, “Mixed” or “Other” |
| 1. Do you participate in an AES? |  |
| 1. How long has your farm been in an AES (any scheme, including previous agreements)? |  |
| 1. Does this include any ditch management options, hedge management options, buffer management options or options for arable land? | Farmers were asked to specify options |
| Questions about specific ditches, indicated on the map |  |
| 1. How long ago was the ditch created? | Farmers were given the option to give the actual number then prompted with “at least 10 years ago”, “at least 20 years ago” or “more than 50 years ago” |
| 1. What would you describe as the primary purpose of the ditch? | Farmers were given the options “drainage”, “boundary definition”, “stock retention”. |
| 1. How often is this ditch wet? | Farmers were given options – “ Dry all year round”, “Wet only after heavy rain”, “Wet only in winter”, “Wet only in winter and after heavy rain in summer”, “Wet all year round”. |
| 1. In the past 25 years, have you (or someone else, e.g. EA or Thames Conservancy) ever carried out the following management on this ditch, and if so when? (If more than one please specify how many times and roughly how often) 2. Vegetation cut? 3. Dredged? 4. Herbicide applied? 5. Increased the width or depth? | Interviewer had table with list of ditches split into two banks. If farmer replied yes to any of the options they were then asked which bank the management was applied to (if appropriate), and if it was the entire length of the bank or channel. |
| 1. Is the ditch pumped? If so when? |  |
| 1. Do you carry out any hedge management, and if so what and when? | Only asked if ditch in question had a hedge |
| 1. What was the field margin sown with (i.e. grass mix, wildflower mix)? | For those ditches with a margin/buffer strip |
| 1. How old are the field margins? |  |
| 14. How often do you cut the field margins and when? |  |
