# Supplementary material for: Enhancing the Biodiversity of Ditches in Intensively Managed UK Farmland
Source: PLoS One. 2015 Oct 7;10(10):e0138306. doi: 10.1371/journal.pone.0138306 (PMC4596843; doi:10.1371/journal.pone.0138306)
Supplement: S2 Table — (DOCX) [file pone.0138306.s003.docx]

Table S2. Full list of models used in the AIC model selection procedure. Three model selection procedures were carried out for bank vegetation data (Berger-Parker Index), channel vegetation data (taxonomic richness) and aquatic invertebrate data (taxonomic richness). Models varied slightly between three response variables (see additional notes).

| Model | Explanatory terms | Hypothesis | Additional information |
| --- | --- | --- | --- |
| 1 | Null model |  | Includes only the random model |
| 2 | Survey area | Reduced survey area may have reduced species richness | For invertebrate surveys, the length surveyed was occasionally less than the standard 50m |
| 3 | Ditch area | Smaller ditches have reduced species richness as the species pool from which the sample is drawn is potentially smaller. |  |
| 4 | Bank angle | Steep banks will have reduced species richness due to increased shading (but opposite effects have been found see [[1](#_ENREF_1)]) | Average for invertebrate data and channel vegetation data, individual values for bank vegetation data |
| 5 | Average water depth | Increased water depth will result in increased species richness [[2](#_ENREF_2)] |  |
|  | Aspect | South facing ditch banks may have greater species richness due to higher levels of insolation[[1](#_ENREF_1)] | Bank vegetation data set only |
| 6 | pH | Low pH levels result in reduced invertebrate species richness (e.g. [[3](#_ENREF_3)]) | Only for Invertebrate analyses. Not used for channel vegetation data as dry ditches therefore excluded |
| 7 | Conductivity | High conductivity results in reduced species richness, as it can indicate high levels of total dissolved solids including pollutants such as urea. | Only for Invertebrate analyses |
| 8 | Nitrogen | High nitrate content results in reduced species richness as nutrient sensitive species decline [[2](#_ENREF_2),[4](#_ENREF_4)]. | Only for Invertebrate analyses |
| 9 | Phosphate | High phosphate content results in reduced species richness due to loss of pollution intolerant species [[5](#_ENREF_5)]. | Only for Invertebrate analyses |
| 10 | Percent arable | A high proportion of arable land surrounding ditch may result in increased sediment runoff and increased agricultural pollution resulting in reduced species richness [[6](#_ENREF_6)]. |  |
| 11 | AES ditch management options selected at the farm level | AES ditch options applied anywhere on the farm lead to more environmentally sensitive ditch management and therefore increased species richness. |  |
| 12 | Ditch in AES | Environmentally sensitive ditch management results in an increase in species richness in these ditches (but see [[7](#_ENREF_7)]) |  |
| 13 | Time since dredged | An increase in the time since dredged leads to an increase in species richness as communities recover from disturbance [[5](#_ENREF_5)]. |  |
| 14 | Time since dredged + Time since dredged^2^ | The relationship between time since dredged and species rich is quadratic with an initial increase in species richness after dredging followed by a decline as dominant species take over [[2](#_ENREF_2),[8](#_ENREF_8)]. |  |
| 15 | Time since vegetation cut | An increase in the time since vegetation cut leads to an increase in species richness as communities recover from disturbance [[9](#_ENREF_9)]. |  |
| 16 | Time since vegetation cut + Time since vegetation cut^2^ | The relationship between time since vegetation cut and species richness is quadratic with an initial increase in species richness after mowing followed by a decline as dominant species take over. |  |
| 17 | Percent shade over channel | As the percent shade over the channel increases species richness increases as high shade levels lead to reduced temperature fluctuations [[10](#_ENREF_10)]. |  |
| 18 | Percent shade over channel + Percent shade over channel^2^ | The relationship between the amount of shade over the channel and species richness is quadratic with an initial increase as shade leads to reduced temperature fluctuations but high levels of shade lead to a loss of macrophytes. |  |
| 19 | Total margin width | Greater total margin width results in an increase in species richness as margins reduce the amount of agricultural pollutants entering the ditch[[11](#_ENREF_11)]. |  |
| 20 | Percent hedge | An increase in the amount of hedge next to a ditch may protect the ditch from agrochemicals [[12](#_ENREF_12),[13](#_ENREF_13)] and reduce temperature fluctuations leading to an increase in species richness. |  |
| 21 | Percent arable +Total margin width | An increase in arable land may lead to increased agricultural pollutants and reduced species richness and having a field margin may have a positive effect on species richness. |  |
| 22 | Percent arable *Total margin width | The effect of increased arable land on a ditch is mitigated by the presence of a field margin [[14](#_ENREF_14)]; those ditches surrounded by a high proportion of arable land but with a margin will have greater species richness than those with a high proportion of arable land but without a margin. |  |
| 23 | Percent hedge+ Total margin width | Both increased amount of hedge and increased amount of margin result in an increase in species richness in the ditch. |  |
| 24 | Percent hedge* Total margin width | The amount of margin around a ditch interacts with the amount of hedge, and a ditch with both hedge and margin will have greater species richness due to positive effects of both. |  |
| 25 | Percent arable *Percent hedge*total margin width | The positive effects of having a hedge and margin will be greater in ditches which are surrounded by a high proportion of arable land. |  |
| 26 | Spatial connectivity | An increase in spatial connectivity to other ditches results in an increase in species richness as allows increased colonisation from other ditches. |  |
| 27 | Temporal connectivity | Ditches with greater temporal connectivity (i.e. wetter for a longer time) have higher species richness as they include groups which are sensitive to water fluctuations [[15](#_ENREF_15)] | For invertebrate surveys, two categories, temporary and permanent, for plant analyses four categories (see main text for details). |
| 28 | Spatial connectivity + Temporal connectivity | Increased spatial connectivity and increased temporal connectivity have a positive effect on species richness, as they improve colonisation rates. |  |
| 29 | Spatial connectivity*Temporal connectivity | Spatial connectivity will have a greater effect on species richness in ditches with low temporal connectivity as it will increase colonisation rates following dry periods [[16](#_ENREF_16)]. |  |
| 30 | Ditch area *Spatial connectivity | Small ditches which also have high spatial connectivity are likely to have greater species richness than small ditches with low spatial connectivity, due to increased ability of species to colonise well connected ditches. |  |
| 31 | Ditch Option*Spatial Connectivity | Environmentally friendly ditch management can mitigate for lack of spatial connectivity by preventing disturbance across entire ditch |  |
| 32 | Ditch Option*Temporal connectivity | Environmentally sensitive management can mitigate for reduced temporal connectivity by preventing disturbance across the entire ditch |  |
| 33 | Dredged*Connectivity per ditch*Temporal Connectivity | Ditches with low levels of spatial and temporal connectivity which have been dredged are likely to have low levels of species richness as recolonization rates are reduced. |  |
| 34 | Dredged* spatial connectivity | Ditches with low spatial connectivity which have been recently dredged are likely to have lower species richness as low spatial connectivity reduces colonisation rates. |  |
| 35 | Dredged*Temporal connectivity | Ditches with low temporal connectivity which have been recently dredged are likely to have lower species richness as low temporal connectivity reduces colonisation rates |  |
| 36 | Ditch Option * Spatial connectivity *Temporal connectivity | Environmentally sensitive management can alter the effects of spatial and temporal connectivity on species richness by reducing the level of disturbance in a ditch. |  |
| 37 | Global model |  | Includes all main terms and interactions in smaller models |

References

1. Van Strien A, Van Der Linden J, Melman TC, Noordervliet M (1989) Factors affecting the vegetation of ditch banks in peat areas in the western Netherlands. Journal of Applied Ecology: 989-1004.

2. Twisk W, Noordervliet MAW, ter Keurs WJ (2003) The nature value of the ditch vegetation in peat areas in relation to farm management. Aquatic Ecology 37: 191-209.

3. Courtney L, Clements W (1998) Effects of acidic pH on benthic macroinvertebrate communities in stream microcosms. Hydrobiologia 379: 135-145.

4. Blomqvist MM, Vos P, Klinkhamer PGL, ter Keurs WJ (2003) Declining plant species richness of grassland ditch banks--a problem of colonisation or extinction? Biological Conservation 109: 391-406.

5. Twisk W, Noordervliet MAW, ter Keurs WJ (2000) Effects of ditch management on caddisfly, dragonfly and amphibian larvae in intensively farmed peat areas. Aquatic Ecology 34: 397-411.

6. Tarmi S, Tuuri H, Helenius J (2002) Plant communities of field boundaries in Finnish farmland. Agricultural and Food Science in Finland 11: 121-135.

7. Blomqvist MM, Tamis WLM, de Snoo GR (2009) No improvement of plant biodiversity in ditch banks after a decade of agri-environment schemes. Basic and Applied Ecology 10: 368-378.

8. Van Strien AJ, Van Der Burg T, Rip WJ, Strucker RCW (1991) Effects of mechanical ditch management on the vegetation of ditch banks in Dutch peat areas. Journal of Applied Ecology 28: 501-513.

9. Milsom TP, Sherwood AJ, Rose SC, Town SJ, Runham SR (2004) Dynamics and management of plant communities in ditches bordering arable fenland in eastern England. Agriculture, Ecosystems & Environment 103: 85-99.

10. Blann K, Nerbonne JF, Vondracek B (2002) Relationship of riparian buffer type to water temperature in the driftless area ecoregion of Minnesota. North American Journal of Fisheries Management 22: 441-451.

11. Musters CJM, van Alebeek F, Geers RHEM, Korevaar H, Visser A, et al. (2009) Development of biodiversity in field margins recently taken out of production and adjacent ditch banks in arable areas. Agriculture, Ecosystems & Environment 129: 131-139.

12. Grimaldi C, Fossey M, Thomas Z, Fauvel Y, Merot P (2012) Nitrate attenuation in soil and shallow groundwater under a bottomland hedgerow in a European farming landscape. Hydrological Processes 26: 3570-3578.

13. Cuttle SP, Macleod CJA, Chadwick DR, Scholefield D, Haygarth PM, et al. (2007) An Inventory of Methods to Control Diffuse Water Pollution from Agriculture (DWPA): User Manual Aberystwyth: IGER/ADAS.

14. Borin M, Bigon E, Zanin G, Fava L (2004) Performance of a narrow buffer strip in abating agricultural pollutants in the shallow subsurface water flux. Environmental Pollution 131: 313-321.

15. Collinson NH, Biggs J, Corfield A, Hodson MJ, Walker D, et al. (1995) Temporary and permanent ponds: An assessment of the effects of drying out on the conservation value of aquatic macroinvertebrate communities. Biological Conservation 74: 125-133.

16. Frisch D, Cottenie K, Badosa A, Green AJ (2012) Strong Spatial Influence on Colonization Rates in a Pioneer Zooplankton Metacommunity. PLoS ONE 7: e40205.
